# Endoscopic supracerebellar infratentorial approach to pineal region epidermoid tumor

**DOI:** 10.3171/2021.4.FOCVID2131

**Published:** 2021-07-01

**Authors:** John Muse, Luke Silveira, Adam Olszewski, Erin D’Agostino, Bruce Tranmer, Brandon Liebelt

**Affiliations:** Department of Neurosurgery, University of Vermont Medical Center, Burlington, Vermont

**Keywords:** pineal epidermoid, paramedian, supracerebellar infratentorial

## Abstract

Epidermoid cysts of the pineal region are a rare entity. Herein, the authors describe the endoscopic resection of a recurrent pineal region epidermoid by way of a supracerebellar infratentorial approach. The patient was positioned in the semiseated upright position with head tilted to the right and slightly flexed, maximizing gravity-based cerebellar retraction, and a paramedian craniotomy was performed owing to the gradual flattening of the tentorium from medial to lateral. This setup, in tandem with the enlarged viewing window achieved by use of 0°, 30°, and 70° endoscopes, afforded the necessary access to achieve a satisfactory resection through this anatomical corridor.

The video can be found here: https://stream.cadmore.media/r10.3171/2021.4.FOCVID2131.

## Transcript

**0:22** This video depicts an endoscopic supracerebellar infratentorial approach to pineal region epidermoid tumor.

The patient is a 51-year-old woman with a prior history of a left parietal craniotomy for resection of a primarily lateral ventricular epidermoid tumor. The tumor recurred at the deep aspect of the cavity in the pineal region with symptoms of visual difficulties and worsening headaches.

Imaging revealed a mass with diffusion restriction centered in the pineal region with some extension into the supratentorial space.

**0:54** The patient was positioned in semisitting position in preparation for a left paramedian suboccipital craniotomy. The head was turned to the left and flexed to expose the left parietal-occipital region. A craniotomy was planned with a linear incision one-third above and two-thirds below the transverse sinus.^1,2^

Prior to surgery, the patient underwent a transthoracic echocardiogram with bubble study to rule out a PFO.

**1:20** Key components of the positioning are depicted here. The head is flexed to flatten the steep angle of the tentorium. The head is rotated to the left in order to provide a more head-on, direct approach given the paramedian craniotomy location. A paramedian craniotomy is selected for supracerebellar infratentorial approaches given the steep angle of the tentorium at the level of the midline, which naturally flattens out as you move more laterally.^3–5^

**1:49** After the craniotomy is performed exposing the transverse sinus, the dura is then flapped superiorly in order to provide maximal viewing angles over the surface of the cerebellum with direct line-of-sight access along the tentorium to minimize any retraction on the cerebellum.

The endoscope is held by the assistant and placed at the corner of the craniotomy during the case. We prefer this setup to a fixed endoscope holder, as it provides dynamic movement to adjust the field of view quickly as the operation dictates.

**2:07** The endoscope is brought into the field. Telfa patties are utilized to pad the superior surface of the cerebellum for the approach. Bridging veins are identified and cauterized and then cut away from the tentorium in order to prevent additional bleeding. We continue to approach the pineal region, gently dissecting the cerebellum away from the tentorial surface. The beginning of the tumor is visualized. The midline tethering adhesions are identified and cauterized and cut in order to further mobilize the cerebellum and allow gravity-assisted retraction to be maximized.

**3:12** We then continue along our approach to the tumor and continue to cauterize and cut small vessels at the border of the tumor. Additional microdissection is performed to release the cerebellum from the underlying epidermoid tumor.

**3:29** The pearly white nature of the tumor is visualized as the tumor comes into view. Further microdissection frees the edge of the cerebellum from the tumor. Hemostasis is meticulously achieved throughout the operation given the depth of the operative approach and the eloquent nature of the surrounding structures. Further dissection with Spetzler dissectors is utilized to free the tumor from the tentorium as well as the surrounding cerebellum, and the tumor is removed in piecemeal fashion with pituitary rongeurs, suction, and microdissectors.

**4:07** After the planes are developed well within the tumor, we begin to debulk the tumor from the inside, removing the tumor as it becomes freed from the surrounding structures.

**4:23** Pituitary rongeurs are useful for the piecemeal resection. Spetzler dissectors are also very beneficial during the dissection, as they can be used to free up fragments of tumor. The 30° endoscope is then inserted in order to visualize around corners, which were not readily apparent with the 0° endoscope. We then continue the resection of this area of tumor with angled instruments.

**4:59** We begin to see normal surrounding neural tissue at the edge of the resection cavity. Additional tumor is visualized at the anterior and superior aspects of the cavity, which is removed with suction and curettes. We continue to define the boundaries of the tumor and visualize the normal underlying neural parenchyma.

**5:21** We then insert a 70° scope in order to further visualize tumor, which was hidden beyond the confines of the normal viewing angle that a microscope would provide. This superior visualization is one of the major advantages of utilizing endoscopes in this type of surgery. Angled Spetzler dissectors are continually used throughout the resection in order to reach around corners and free up the tumor from the surrounding brain.

**5:51** Tumor is gently dissected away from critical vascular structures deep within the cavity with Rhoton dissectors and removed in piecemeal fashion. The 70° scope is then used to look superiorly in the supratentorial compartment to continue to remove tumor that extended up to the prior resection bed.

**6:23** Here, we are looking into the left lateral ventricle where the tumor previously was resected from. We are now looking into the lateral ventricle where the prior tumor resection was performed. We can see into the frontal and temporal horns and directly into the atrium.

**6:46** Continued tumor resection is performed until an excellent resection is achieved.

We then inspect the boundaries and extents of the cavity with neuronavigation to ensure maximal resection of the tumor.

Small areas of tumor capsule which are densely adherent to the underlying pineal region and tectum are left undisturbed to prevent neurological deficit.

**7:13** The surgical cavity is lined with Surgicel for hemostatic purposes.

**7:17** As we remove the Telfa patties, we see the superior surface of the cerebellum had been well protected throughout the surgery without retraction injury.

**7:25** Postoperative imaging shows the excellent resection of the tumor without complication. A small focus of restricted diffusion is present near the left temporal horn which was not easily accessible from this approach.

**7:38** The patient did well postoperatively with a transient Parinaud syndrome, which resolved over the course of the first few days during her inpatient stay.

## Author Contributions

Primary surgeon: Liebelt. Assistant surgeon: Olszewski, Tranmer. Editing and drafting the video and abstract: Liebelt, Muse, Silveira, Olszewski. Critically revising the work: Liebelt, Muse, Olszewski, D’Agostino, Tranmer. Reviewed submitted version of the work: Liebelt, Silveira, Olszewski, D’Agostino. Approved the final version of the work on behalf of all authors: Liebelt. Supervision: Liebelt. Illustrator: D’Agostino.
